# Of Mice and Men: The Coronavirus MHV and Mouse Models as a Translational Approach to Understand SARS-CoV-2

**DOI:** 10.3390/v12080880

**Published:** 2020-08-12

**Authors:** Robert W. Körner, Mohamed Majjouti, Miguel A. Alejandre Alcazar, Esther Mahabir

**Affiliations:** 1 Department of Pediatrics, Faculty of Medicine and University Hospital Cologne, University of Cologne, 50937 Cologne, Germany; robert.koerner@uk-koeln.de; 2 Comparative Medicine, Center for Molecular Medicine Cologne (CMMC), University of Cologne, Faculty of Medicine and University Hospital Cologne, 50931 Cologne, Germany; mohamed.majjouti@uk-koeln.de; 3 Department of Pediatric and Adolescent Medicine, Translational Experimental Pediatrics—Experimental Pulmonology, Faculty of Medicine and University Hospital Cologne, University of Cologne, 50937 Cologne, Germany; miguel.alejandre-alcazar@uk-koeln.de; 4 Center for Molecular Medicine Cologne (CMMC), Faculty of Medicine and University Hospital Cologne, University of Cologne, 50931 Cologne, Germany; 5 Member of the German Center for Lung Research (DZL), Institute for Lung Health, University of Giessen and Marburg Lung Center (UGMLC), 50937 Cologne, Germany

**Keywords:** COVID-19, SARS-CoV-2, mouse hepatitis virus (MHV), viral infection

## Abstract

The fatal acute respiratory coronavirus disease 2019 (COVID-19) is caused by severe acute respiratory syndrome coronavirus 2 (SARS-CoV-2). Since COVID-19 was declared a pandemic by the World Health Organization in March 2020, infection and mortality rates have been rising steadily worldwide. The lack of a vaccine, as well as preventive and therapeutic strategies, emphasize the need to develop new strategies to mitigate SARS-CoV-2 transmission and pathogenesis. Since mouse hepatitis virus (MHV), severe acute respiratory syndrome coronavirus (SARS-CoV), and SARS-CoV-2 share a common genus, lessons learnt from MHV and SARS-CoV could offer mechanistic insights into SARS-CoV-2. This review provides a comprehensive review of MHV in mice and SARS-CoV-2 in humans, thereby highlighting further translational avenues in the development of innovative strategies in controlling the detrimental course of SARS-CoV-2. Specifically, we have focused on various aspects, including host species, organotropism, transmission, clinical disease, pathogenesis, control and therapy, MHV as a model for SARS-CoV and SARS-CoV-2 as well as mouse models for infection with SARS-CoV and SARS-CoV-2. While MHV in mice and SARS-CoV-2 in humans share various similarities, there are also differences that need to be addressed when studying murine models. Translational approaches, such as humanized mouse models are pivotal in studying the clinical course and pathology observed in COVID-19 patients. Lessons from prior murine studies on coronavirus, coupled with novel murine models could offer new promising avenues for treatment of COVID-19.

## 1. Introduction

In December 2019, a newly identified β-coronavirus infected thousands of people in the Wubei province, China, causing the acute respiratory coronavirus disease 2019 (COVID-19) (https://globalbiodefense.com/novel-coronavirus-covid-19-portal/). COVID-19 is a highly transmittable and potentially fatal viral infection caused by the severe acute respiratory syndrome coronavirus 2 (SARS-CoV-2). The outbreak originating in China spread worldwide, caused major socio-economic and health consequences and was declared a pandemic by the World Health Organization (WHO) on 11 March 2020 [1]. This pandemic has, in particular, exposed vulnerable populations to a global health crisis. As of 30 June 2020, over 10 million people were tested positive for SARS-CoV-2, which pushed the health system in various countries to its limits and resulted in more than 500,000 deaths worldwide [2]. To date, there are neither proven options for prophylaxis nor for therapy. The steadily increasing numbers of infected persons are alarming and urge deciphering the pathomechanisms of COVID-19 to define new tools for risk stratification and development of novel treatment strategies. Comprehensive studies, including clinical and experimental approaches are of paramount importance.

For decades, the mouse has served as an excellent model not only to investigate inflammation, immune response, and infections including those of a viral nature, but also to develop new diagnostic, preventive, and therapeutic approaches. Infection models comprise various viruses including the respiratory or enterotropic mouse hepatitis virus (MHV), which belongs to the coronavirus family of enveloped positive-strand RNA viruses. Since SARS-CoV-2 is a coronavirus, the murine infection with MHV amongst others could serve as an experimental model to study principles of COVID-19. The present review provides a comprehensive overview of coronavirus in mice and the newly discovered SARS-CoV-2, putting these viruses into relation to other coronaviruses. Integration of murine expertise in viral infection could offer the opportunity to derive new strategies to rapidly decipher the pathomechanisms of COVID-19. Here, we focused on major topics that comprise a description of coronaviruses, host species as well as organotropism, transmission, clinical disease, pathogenesis, therapy, and control of MHV and COVID-19. We also provide information on the relevance of MHV and mice as models for widening the knowledge of the pathogenesis and therapeutic approaches for the human coronaviruses with the emphasis on SARS-CoV-2.

## 2. Coronaviruses

The family Coronaviridae belongs to the suborder Cornidovirineae, which is one of eight suborders forming the order Nidovirales. It consists of the two sub-families Letovirinae and Orthocoronavirinae. Based on genetics and serology, Orthocoronavirinae comprise the four genera Alpha-, Beta-, Gamma-, and Deltacoronavirus. The genus Betacoronavirus is made up of four subgenera. The subgenus Embecovirus contains the mouse hepatitis virus (MHV) and the subgenus Sarbecovirus includes SARS-CoV and SARS-CoV-2 [3]. Coronavirinae virions are pleomorphic or spherical, 80-220 nm in diameter, enveloped, and have large club-shaped spikes (peplomers). The genome consists of a single molecule of linear positive-sense, single-stranded RNA, which is 25-31 kb in size. Viruses replicate in the cytoplasm. The virions contain four or five structural proteins, which are a major spike glycoprotein (S), an envelope protein (E), a membrane protein (M), a nucleoprotein (N), and, in some viruses, a hemagglutinin esterase (HE) [4]. Additionally, coronaviruses encode a variable number of accessory proteins, which are able to modulate virus-host interactions and thereby influence pathogenesis [5]. For example, the accessory proteins encoded by SARS-CoV open reading frames 3b and 6 are antagonists of the innate immune system, interfering with the development of type I interferon responses [6]. Coronaviruses have a vast genetic diversity due to point mutations by polymerase errors. Moreover, genetic recombinations occur frequently between the genomes of different but related coronaviruses during co-infections. These mechanisms allow constant generation of new viruses with novel phenotypes, which differ with respect to host and tissue tropism, virulence, and the resulting disease characteristics [5]. By population genetic analyses of 103 SARS-CoV-2 genomes, two major variants of SARS-CoV-2 were defined by two single nucleotide polymorphisms (SNPs), which show nearly complete linkage across the analyzed strains. Among these, the so-called “L type” named after the amino acid leucine was more prevalent (~70%) than the so-called “S type” (~30%, named after the amino acid serine). The S type was found to be the evolutionary older version.

### Host Species

Coronavirus infections have been described in various species such as pigs, cattle, camels, cats, dogs, rodents, birds, and bats. Middle East Respiratory Syndrome (MERS)-CoV, SARS-CoV, and SARS-CoV-2 are zoonoses [5,7,8]. Since SARS-CoV-2 viruses isolated from numerous patients have a sequence identity higher than 99.9%, a very recent host shift into humans was suggested [8]. It is primarily the S protein, which determines host spectrum and tropism of individual coronaviruses. The S protein of SARS-CoV-2 is composed of the S1 subunit, which contains the RBD and the S2 subunit, which mediates fusion between the viral and host cell membrane [9]. The structural similarity of the RBD undermines the evolutionary relationship between SARS-CoV and SARS-CoV-2 [10]. In addition to receptor binding, virus fusion by the action of host cell-specific proteases, which prime the S protein, appears to be a further way of regulating coronavirus infection, as well as host and tissue tropism [5]. The SARS-CoV-2 S protein harbours a furin cleavage site at the boundary of the S1 and S2 subunits, which is a unique difference, compared to SARS-CoV. It might be possible that the almost ubiquitous expression of furin-like proteases could enhance cell and tissue tropism of SARS-CoV-2, thereby increasing its transmissibility and altering its pathogenicity [9]. Coronaviruses make use of different cellular proteins as receptors. During the adaptation of SARS-CoV to humans, minimal genetic changes affected the S gene, allowing binding to the human angiotensin converting enzyme 2 (ACE2) receptor, and it seems that a similar process occurred in SARS-CoV-2 [10]. The adaptation might have occurred due to recombination or, more likely, to natural selection either in an animal host before zoonotic transfer or in humans following zoonotic transfer [11]. SARS-CoV-2 also utilizes the ACE2 receptor and strongly binds to the human and bat type [9,12,13]. It does not bind to the murine ACE2 receptor [10,13]. Among all coronaviruses, MHV is unique since it uses the N-terminal domain of its spike protein to bind to the carcinoembryonic antigen-related cell adhesion molecule 1 (CEACAM-1a) receptor [14].

As with SARS-CoV, SARS-CoV-2 was first described in persons who were exposed to a live-animal market in China [15]. In the case of SARS-CoV SARS-like coronaviruses were isolated from Himalayan palm civets (Paguma larvata) and a raccoon dog (Nyctereutes procyonoides) from live-animal markets but not in the wild. It was suspected that civets and raccoon dogs served as intermediate hosts but bats were proposed to be the natural reservoir hosts of SARS-CoV. SARS-like coronaviruses with a broad genetic spectrum were isolated from Chinese horseshoe bats (Rhinolophus sinicus). SARS-CoV-2 is 96% identical at the whole genome level to a bat coronavirus (RaTG13) and shares 79.6% sequence identity to SARS-CoV [13]. Apart from the strong evidence that SARS-CoV-2 also originated in bats [10,13], it remains unclear how the bat-human transmission occurred. Pangolins have been considered as potential intermediate hosts [16]. Interestingly, SARS-CoV-2-related coronaviruses in pangolins show an 85.5% to 92.4% sequence similarity to SARS-CoV-2 at the whole genome level [16] and a 97.4% amino acid similarity in the RBD of SARS-CoV-2. However, the remainder of the genome of SARS-CoV-2 is more closely related to the bat coronavirus RaTG13 [16]. As with SARS-CoV, the RaTG13 and the examined pangolin coronaviruses also lack the furin-like cleavage site in the S protein. This polybasic cleavage site might have facilitated the rapid spread of SARS-CoV-2 in the human population [16]. After all, the exact route of transmission from natural reservoirs to humans remains speculative.

## 3. Coronaviruses in Mice

MHV is a natural pathogen of mice (*Mus musculus*) and refers to many named and unnamed strains of murine coronavirus. It was first isolated in 1949 [17]. MHV has been extensively used as a model coronavirus to study hepatitis [18] and demyelinating diseases such as multiple sclerosis [19] in humans. Since multiple strains exist, MHV differs in organotropism, virulence, and pathogenicity.

### 3.1. Organotropism

MHV strains possess a primary tropism for either the upper respiratory or enteric mucosa. The respiratory (polytropic) MHV strains MHV-1, MHV-2, MHV-3, MHV-JHM (MHV-4), MHV-A59, and MHV-S initially replicate in the nasal respiratory and olfactory epithelium, with subsequent viremia and dissemination to the lung, liver, bone marrow, brain, lymphoid tissue, and reproductive organs [20,21,22,23,24,25]. The enterotropic strains, including MHV-D, MHV-DVIM, MHV-Y, and MHV-RI, are restricted largely to the intestine with excretion primarily in feces and can spread to the liver, lymphoid tissue, and spleen [26,27].

### 3.2. Transmission

MHV is highly contagious in laboratory mice, having been one of the most prevalent viruses in mouse colonies worldwide two decades ago [25]. The duration of infection varies depending on the MHV strain, route of inoculation as well as host factors including age, immunocompetence, passive immunity, genetic strain, and genetic alterations [22,24,28]. Enterotropic MHV is shed in very high titers in feces [24,29]. Virus clearance commences approximately one week after infection with virus elimination in most mice within 3-4 weeks [20,22,28]. Therefore, the virus can be transmitted orally to cage mates for a period of 30 days after infection [24,30]. Transmission of respiratory MHV occurs via direct contact with infected mice. An infection with MHV spreads rapidly when the virus is introduced into a naïve colony kept in open as opposed to micro-isolator cages, which offer biocontainment. Oronasal inoculation of pregnant mice with MHV showed transmission in utero, which depended upon the MHV strain and the host genotype. Virulent, polytropic MHV-JHM was recovered from the uterus and placenta as well as from fetuses during all three trimesters of pregnancy in susceptible BALB/cByJ mice while low virulent, polytropic MHV-S was detected at a low percentage, and enterotropic MHV-Y was not found in any fetuses [31].

### 3.3. Clinical Disease

Less virulent strains include MHV-1, MHV-S, MHV-Y, and MHV-Nu. Others such as MHV-2, MHV-3 as well as MHV-A59 are more virulent and MHV-JHM is neurovirulent [17,32,33,34,35]. The symptoms of MHV infection in experimental studies range from subclinical manifestations in adult mice to high morbidity and mortality in neonatal or young mice, depending on the virus strain, route of infection, genotype, age, and immune status of the host [24]. In immunocompetent adults, MHV infection is inapparent, does not persist, and usually all animals are infected. In neonates, diarrhea and a death rate close to 100%, due to severe enteritis, malabsorption, and dehydration are observed [24,28,36]. In immunodeficient mice, a wasting disease accompanied by chronic weight loss with non-pathogenic MHV strains or acute death with more virulent strains occurs [27]. Flaccid paralysis of hind limbs in suckling mice has also been reported [17].

### 3.4. Pathogenesis

The pathogenesis of MHV is well-studied. In polytropic strains, there is mild necrosis of the nasal epithelium, pneumonia, necrosis as well as syncytia formation in the spleen, lymph nodes, Peyer’s patches, thymus, liver, and bone marrow as well as necrotizing encephalitis with demyelination after haematogenous dissemination to the central nervous system (CNS) [17,27]. In enterotropic strains, pathological changes are primarily restricted to the intestinal mucosa and are most severe in infant mice. These include villus attenuation, enterocytic syncytia, and mucosal necrosis [27]. An infection with MHV-3 leads to hepatitis, which is caused, in part, by overexpression of prothrombinase [37].

MHV causes hepatitis, which results in elevated liver enzyme levels and altered patterns of protein synthesis [38,39]. MHV infection increases iron uptake due to tissue injury in the liver [40] and causes changes in blood cells such as anemia, thrombocytopenia, leukopenia, and pancytopenia as well as increased monocyte procoagulant activity that leads to thrombosis, resulting from microcirculatory disturbance [18,41].

MHV is strongly immunomodulating. It causes immunodepression or immunostimulation, depending on the time of infection and circulating interferon [42]. The virus replicates in macrophages [43,44,45], enhances peritoneal macrophage numbers, cytotoxicity in macrophages [46,47], and dysfunction of B- and T-cells [48,49,50,51]. It also reduces spleen cell numbers [52] and activates natural killer cells [53]. In addition, it alters the interferon responsiveness of infected mice [42,53] and reduces the levels of cytokines as well as gamma interferon in splenic cells [49]. After recovering from MHV-A59 infection, there is a permanent decrease of skin graft rejection [54]. When re-infected with different strains of MHV recovered mice have complete or partial protection against T-cell dysfunctions [23]. MHV also induces polyclonal B lymphocyte activation [55]. In chronically MHV-JHM-infected mice, TNF-α, IL-1β, and IL-6 are produced by astrocytes in spinal cords [56]. The cytokines MCP-1, MCP-3, MIP-1β, MIP-2, and RANTES increase on day 7 after infection in brain and spinal cord tissue concomitant with acute viral encephalomyelitis leading to chronic demyelination [57]. To protect the host during MHV-JHM-induced encephalomyelitis, effective coordination of pro- and anti-inflammatory cytokines is essential [58]. MHV leads to neurogenic bladder overactivity caused by demyelination of the CNS similar to that observed in patients with multiple sclerosis [59], hind limb paralysis, and wasting [57,60]. Specific monoclonal antibodies directed against the E2 glycoprotein (now called MHV spike protein S) prevent lethal encephalomyelitis and lead to a demyelinating disease [34]. The anti-MHV-JHM CD4+ and CD8+ T-cells are able to mediate demyelination [57,61].

### 3.5. Therapy and Control

MHV infection is usually self-limiting in immunocompetent mice but can persist in immunodeficient mice. Seroconversion of MHV-infected mice generally occurs between day 7 and day 20 after infection [20,24,33,62,63]. Previous reports showed that pups obtain colostral MHV-specific IgA and IgG antibodies from their MHV-seropositive mothers [25,31,64]. These maternal antibodies protect them against MHV infections for approximately 3-4 weeks. After this period, the maternal antibodies decline [31,64]. Immunocompetent mice which recover from an infection with MHV are resistant to strain-specific re-infection [65,66,67]. As practiced under routine conditions, serology can be performed to test whether mice produced antibodies.

To prevent the spread of MHV in a mouse colony, breeding is cessated, existing breeding pairs are separated [68], and no naïve mice are introduced into the colony. In the laboratory animal field, mice that are naturally infected do not undergo therapy but are usually culled and rederived via embryo transfer [69].

Vaccines are not used to control MHV, due to the strain specificity of immunity, high mutation rate of the virus, and interference of research. However, MHV can be used as an experimental model for vaccine development for other coronaviruses [27].

## 4. Coronavirus in Humans

### 4.1. Organotropism

In general, the receptor specificity primarily determines the permissivity of cells, which, in turn, determines clinical disease. Since SARS-CoV-2 binds to the ACE2 receptor, based on the knowledge of SARS, organs seem to be affected depending on the level of ACE2 expression. Notably, ACE2 expression in airway epithelial cells and enterocytes determines clinical symptoms and plays an important role in transmission [70]. ACE2 expression is highest in nasal mucosa since it contains the highest percentage of ACE2-expressing ciliated cells. It gradually decreases throughout the lower respiratory tract from large to small airways. Accordingly, infectivity of airway epithelial cells follows the same pattern leading to highest viral loads in the nose [71]. Moreover, ACE2 is abundantly present in the human body including the heart, kidney, liver, gallbladder, esophagus, testis, the endothelial cells of arteries and veins as well as arterial smooth muscle cells [72,73,74,75]. ACE2 has also been identified in the cornea and conjunctiva and ocular symptoms are common in COVID-19, sometimes appearing before the onset of respiratory symptoms [76]. Therefore, the eyes also pose a possible route of infection.

Regarding SARS-CoV, post-mortem examination confirmed SARS-CoV RNA in the heart, kidney, liver, and spleen. However, the highest RNA concentrations were found in the small intestine and the lung pointing to a specific tropism for these organs [77]. In autopsies of SARS-CoV patients, histopathological changes were also found in the cortex and hypothalamus of the brain [78]. In mice, SARS-CoV was detected in the olfactory bulb and in the brain stem after inhalation of the virus [79]. Since patients with COVID-19 occasionally present with confusion, headache, anosmia, and ageusia the question whether there is a neurotropism of SARS-CoV-2 has also been raised [80].

In the lung, overall alveolar expression of ACE2 is lower than in bronchi [71]. Infection of the lung might be facilitated in vivo because of a thinner respiratory tract lining fluid of the alveolar epithelium, which makes the ACE2 receptors more accessible [81,82]. SARS-CoV-2 also infects type I pneumocytes and alveolar macrophages and has a similar cell tropism as SARS-CoV [83]. In contrast, MERS-CoV binds to the Dipeptidyl peptidase-4 (DPP4) receptor and is able to infect even a broader range of cells [84]. This is what makes this polytropic coronavirus particularly dangerous and paves the way for zoonotic transfer [5]. Apart from ACE2, evidence for alternative receptors is emerging. For example, SARS-CoV and SARS-CoV-2 are able to bind to cluster of differentiation 209L (CD209L) and cluster of differentiation 209 (CD209). CD209L is markedly expressed in alveolar cells, proximal renal tubular epithelial cells, pulmonary capillaries, and vascular endothelia. It mediates SARS-CoV-2 entry and infection. This mode of infection may contribute to vascular pathology and thrombosis in COVID-19 patients [85,86]. Also, two other proteins CD147 and GRP78 were recently reported to aid in cell entry in the respiratory mucosa [87].

Apart from receptor binding, fusion-activating proteases greatly influence coronavirus virulence [88]. Coronaviruses make use of diverse proteases for cellular entry, for example, acid-dependent endosomal/lysosomal proteases, furin, and transmembrane protease serine subtype 2 (TMPRSS2) [88]. The previously mentioned polybasic cleavage site at the S1-S2 junction in SARS-CoV-2 differentiates it from SARS-CoV, which may lead to an increased fusion activity in tissues with low density of ACE2 expression. TMPRSS2 shows an even broader distribution than ACE2 [74]. The highest expression of ACE2 and TMPRSS2 was found in nasal secretory cells and ciliated cells [74]. Accordingly, nasopharyngeal swabs yielded higher viral loads than throat swabs making the nasal epithelium a portal for infection and transmission [13]. Therefore, the polybasic cleavage site contributes to the higher contagiousness of SARS-CoV-2 compared to SARS-CoV. Interestingly, the most prevalent secretory cell, that is, the MUC5B+ club cell, is not infected by SARS-CoV-2 despite expression of ACE2 and TMPRSS2. This suggests that there are more factors influencing infectivity of cells apart from the mere presence of specific receptors [71]. Although SARS-CoV-2 has the potential to infect a variety of cells, previous findings demonstrate a predominantly pulmonary tropism making COVID-19 primarily a respiratory disease.

### 4.2. Transmission

SARS-CoV-2 is highly contagious and several factors lead to an increased risk of infection. It spreads from person to person by contact, respiratory droplets, and also by short- and long-range aerosols [13,89,90]. Notably, there is strong evidence for airborne transmission [91]. Even talking and tidal volume breathing bear the risk of exhaling infectious particles, but coughing, sneezing, and close contact considerably increase transmission [91]. SARS-CoV-2 can persist in room air for at least three hours under experimental conditions and its aerosol characteristics are similar to SARS-CoV [92]. The travel distance of virus-carrying particles depends on numerous variables and it is difficult to define a definite safe distance [91]. There is a high viral load in respiratory secretions in the early phase of COVID-19, including the incubation period [93]. During the spread of SARS-CoV, particularly high viral loads in aerosols were correlated to superspreading events [5]. Superspreading events have also been reported for SARS-CoV-2 as recently described in Southeast Asia [94].

SARS-CoV-2 RNA was also detected in the feces of COVID-19 patients including asymptomatic carriers in whom SARS-CoV-2 was no longer detected in pharyngeal swabs, thus enabling fecal transmission by contact or by contaminated surfaces [95,96]. Notably, viable SARS-CoV-2 was detected on contaminated surfaces (plastic and stainless steel) for up to 72 h [92]. Since carriers of SARS-CoV-2 may be asymptomatic and gastrointestinal symptoms may be the only presentation of COVID-19 [97], an infected person might be regarded as healthy by others, resulting in less precaution, which in turn, increases the transmission potential.

Transmission of SARS-CoV-2 by asymptomatic carriers poses a unique risk [98,99]. Tight binding to human ACE2 also increases the efficient transmission of SARS-CoV-2, as it was the case for SARS-CoV [9]. Human placenta highly expresses ACE2 and maternal-fetal transmission by viremia was described recently [100]. However, co-transcription of ACE2 and TMPRSS2 is negligible in the placenta [101].

Apart from the described biological characteristics, the contagiousness is influenced by environmental factors, socio-behavioural factors, living conditions, and political regulations [102]. The often reported basic reproduction (R0) numbers result from complex calculations and strongly depend on the amount of tests conducted, as well as the model used for its estimation. The fact that a considerable proportion of infected people are asymptomatic or show mild disease and therefore are possibly not tested further complicates estimations.

### 4.3. Clinical Disease

The clinical disease is not completely understood and comprises a wide range of symptoms from asymptomatic and mild manifestations to severe and fatal cases [103]. According to a study conducted on a cruise ship, the rate of asymptomatic passengers was estimated to be 18% [104]. The true rate of asymptomatic carriers in the general public is unknown. Symptoms of COVID-19 infection usually appear after an incubation period of approximately 5.2 days [105]. Symptomatic adult patients exhibit predominantly fever, cough, and dyspnea [103]. A further common symptom is fatigue. Pneumonia with bilateral multifocal ground-glass opacities in lung imaging studies is frequently diagnosed, and cases with asymptomatic pneumonia have also been described [105]. Further symptoms comprise sore throat, rhinorrhea, stuffy nose, conjunctivitis, myalgia, vomiting, diarrhea, cephalgia, confusion, ageusia, and anosmia, suggesting that other organ systems may also be affected. In Europe, ageusia and anosmia were reported in over 80% of positive cases [106].

In children, fever is also the most common symptom, followed by dry cough and cephalgia. Acute respiratory distress syndrome (ARDS) and life-threatening multiple organ failure are rarely reported [104]. Children also seem to present more often with gastrointestinal symptoms than adults, including vomiting, abdominal pain, and diarrhea [107]. COVID-19 was found in a few neonates born to SARS-CoV-2-positive mothers. Newborns were symptomatic and showed fever, lethargy, respiratory distress, and pneumonia on chest images [108].

Mild symptoms were reported to decline after approximately a week while severe cases can result in respiratory failure and death. In fatal cases, the period from the onset of COVID-19 symptoms to death ranged from 6 to 41 days with a median of 14 days [109]. In contrast to gastrointestinal symptoms with enterotropic MHV in young mice and COVID-19 in children, elderly people are more susceptible for a severe clinical course than young adults, adolescents, and children, indicating an age-related association with clinical severity. Comorbidities, such as hypertension, diabetes mellitus, cardiovascular disease, chronic respiratory disease, immunosuppression, and cancer increase the risk for severe outcome [110]. In Germany, highest incidences were observed in people older than 90 years (>600/100,000) and 86% of all fatalities were older than 70 years of age. In contrast, only 0.03% of all fatalities were younger than 20 years of age [111]. The underlying biological basis of this age-specific morbidity and mortality is currently unclear.

Morbidity and mortality in COVID-19 are significant. As of 30th June 2020, over 10 million people were tested positive for SARS-CoV-2 and more than 500,000 people died worldwide [2]. The current data result in a case fatality rate (CFR) of 4.95%, which is remarkably higher than that of an influenza infection (typically below 0.1%, influenza season 2018-2019, United States: 0.096%) [112]. However, the CFR is regionally different and depends on many factors, especially the number of tests conducted. Currently, figures for morbidity and mortality are prone to inaccuracies.

### 4.4. Pathogenesis

#### 4.4.1. Viral Entry and Replication

After SARS-CoV-2 makes contact with the respiratory system, it enters and replicates primarily in ciliated respiratory epithelial cells of the upper and lower respiratory tract as well as in type II pneumocytes. In lung pathology, patchy infiltrates indicate aspiration of infectious inocula from the upper respiratory tract [71]. As already mentioned, viral entry also depends on TMPRSS2 activity, which catalyses protein cleavage between the S1 and S2 subunit after the S1 subunit has bound to the ACE2 receptor [113]. Marked gene expression for TMPRSS2 has been reported alongside with ACE2 in type II pneumocytes [114]. The severe infection of type II pneumocytes leads to depletion of progenitors for type I pneumocytes and to a reduced surfactant production, ultimately impairing ventilation and gas exchange [81,115,116].

#### 4.4.2. Immune Evasion

SARS-CoV-2 replicates more efficiently than SARS-CoV in human lung tissues, producing 3.2-fold more infectious virus particles within 48 h after infection [83]. A possible reason for this is the suppression of the innate immune response since SARS-CoV-2 infection does not trigger a significant interferon response and causes a relatively low release of pro-inflammatory cytokines and chemokines at the beginning of the infection [83]. The exact mechanisms of this suppression are unclear. It was postulated that, similar to SARS-CoV, SARS-CoV-2 possesses interferon-antagonising accessory proteins [83]. The immune evasion could be responsible for mild or asymptomatic clinical courses of COVID-19 and could facilitate transmission. Moreover, the accessory proteins of SARS-CoV and SARS-CoV-2 might also be responsible for the increased pathogenicity of these viruses when compared to human coronavirus NL63 (HCoV-NL63). The latter virus also binds to the ACE2 receptor but harbours only a single accessory gene [70].

#### 4.4.3. Cytokine Storm

Ongoing infection with SARS-CoV-2 causes a massive release of pro-inflammatory cytokines and chemokines including IL-6, MCP-1, CXCL1, CXCL5, and CXLC10 (IP-10) in human lung tissue [83]. Blood levels of cytokines and chemokines were elevated including IL-1β, IL-1ra, IL-7, IL-8, IL-9, IL-10, basic FGF2, G-CSF, GM-CSF, IFN-γ, IP-10, MCP-1, MIP-1α, MIP-1β, PDGFβ, TNF-α, and VEGF-A [117]. Inflammation results in an atypical pneumonia and diffuse alveolar damage (DAD) [118,119] comprising alveolar and bronchiolar epithelial necrosis, alveolar edema, hyaline membrane formation, and accumulation of neutrophils, macrophages, and lymphocytes [118,119,120]. The formation of multi-nucleated syncytial cells and atypical enlarged pneumocytes in the intra-alveolar spaces was also described [120]. Clinically, DAD results in ARDS, which is a leading cause of death, and the cytokine storm may also lead to multiple-organ failure [121]. Serum levels of IL-2R and IL-6 in patients with COVID-19 are positively correlated with the severity of the disease. Another finding in COVID-19 that is associated with an adverse outcome is the rapid development of lymphopenia, whereby CD4+ T-cells are more severely reduced than CD8+ T-cells [73,105], the reason being unknown.

The triggering mechanism of the hazardous cytokine storm is still not well-understood, and an unfortunate combination of multiple triggers has been discussed. First, a disturbance in the redox balance activates redox-sensitive transcription factors, such as NF-kB. This condition finally induces pro-inflammatory cytokines including IL-1β, IL-6, and TNF-α, and could thereby account for the activation of pro-inflammatory pathways [122,123]. Second, reduced function of myeloid antigen-presenting cells in the elderly could promote immune evasion of SARS-CoV-2, higher viral loads, and amplified inflammation [124]. Third, antibody-dependent enhancement (ADE) through the interaction with Fc receptors (FcR) and other receptors has been proposed [125,126] to facilitate both persistent inflammatory response and persistent viral replication in the lung [126,127]. Finally, age-related severity may also be related to co-infection with other pathogens that interfere with the interferon-mediated antiviral response or to lacking cross-reactive immunity [73,124].

#### 4.4.4. Adaptive Immune Response

The median seroconversion time for total antibodies to SARS-CoV-2, IgM, and IgG was 11, 12, and 14 days post infection, respectively [128]. Antibodies were present in <40% of patients within the first week after the onset of symptoms and increased to 100% (total antibodies), 94.3% (IgM) and 79.8% (IgG) after day 15 [128]. However, the rise of antibodies was not always accompanied by a reduction of viral RNA, particularly in critically ill patients [128,129]. This finding suggests that antibodies may not be sufficient to clear the virus. A higher titer of total antibodies has also been associated with a worse clinical course [128]. Currently, it is not known how long antibodies to SARS-CoV-2 last or if they protect from re-infection. In the past, it was shown that after infection with SARS-CoV neutralizing antibodies prevent re-infection in animal models [73].

Apart from the formation of antibodies, T-cell immunity is particularly important. In SARS-CoV human memory T-cell responses to the N protein of SARS-CoV persisted for two years in the absence of antigen [73]. In COVID-19 convalescent patients circulating SARS-CoV-2-specific CD8+ and CD4+ T-cells were identified in approximately 70%, and 100%, respectively [124]. CD4+ T-cell responses to S protein correlated with the magnitude of IgG- and IgA-titers. Interestingly, SARS-CoV-2-reactive CD4+ T-cells were also found in 40 to 60% of unexposed individuals suggesting cross-reactive T-cell recognition between other coronaviruses and SARS-CoV-2. This might influence the individual clinical course of COVID-19.

#### 4.4.5. Role of ACE2

Apart from viral entry, ACE2 also seems to play an important role in lung injury. Infection with SARS-CoV-2 leads to a down-regulation of ACE2 on infected cells [130]. In animal models, the loss of ACE2 leads to an increased production of harmful reactive oxygen species (ROS), enhanced vascular permeability, lung edema, and neutrophil accumulation [131,132]. The resulting increase in angiotensin II further enhances lung inflammation and injury by binding to angiotension receptor subtype 1a (AT1aR) [73]. It activates macrophages, leads to the production of pro-inflammatory cytokines such as IL-6 [133], and promotes vasoconstriction and thrombosis [130]. Interestingly, older age, hypertension, diabetes, and cardiovascular diseases share a variable degree of ACE2 deficiency. The additional down-regulation of ACE2 caused by SARS-CoV-2 infection could also explain the increased mortality rate in these patients [130].

SARS-CoV infection leads to an increased release of enzymatically active soluble ACE2 (sACE2) whose function is not fully understood. This effect, also known as “ACE2 shedding”, is not induced by HCoV-NL63, which also binds to the ACE2 receptor. Since HCoV-NL63 just causes the common cold ACE2 shedding was suggested to play a central role in the development of SARS and probably also of COVID-19 [134].

#### 4.4.6. Extrapulmonary Disease

Apart from the respiratory system, SARS-CoV-2 can infect the gastrointestinal system by replicating in enterocytes and leading to increased intestinal permeability and malabsorption [135]. Diarrhea has been described in children [97,108,136]. In severely ill patients, acute cardiac injury in the form of fulminant myocarditis, acute coronary syndrome, and arrhythmias have been described. Further, kidney injury and shock can occur, raising the question of whether there is a specific pathogenic effect of SARS-CoV-2 in other organs or if multi-organ failure occurs due to a systemic inflammatory response [103,137,138]. Inflammation of the vascular system can also result in diffuse microangiopathy with thrombosis, which favors a poor prognosis [138].

### 4.5. Therapy

Currently, there is no specific therapy against COVID-19. In general, therapy consists of supportive measures and intensive care with respiratory support in the severely ill patients. Further supportive measures include fluid replacement therapy, antibiotic therapy, management of comorbidities, and prevention of venous thromboembolism. In SARS-CoV, early treatment with glucocorticoids increased plasma viral load in patients who were not critically ill, leading to an aggravation of the disease and is therefore generally not recommended for COVID-19. On the other hand, in some cases, corticosteroids could prevent the occurrence of ARDS when administered timely in the early stage of the cytokine storm [121]. Therefore, the use of glucocorticoids in critically ill COVID-19 patients is controversial.

Currently, there are various specific therapies under development, and in clinical trials, consisting of drugs that are already approved for other diseases and are being repurposed, which has the advantage of fast availability for off-label use. Therapeutic principles include antivirals, neutralizing antibodies, ACE2 receptor blockers, protease blockers, immunomodulators, surfactant, and vaccination.

As an example of specific therapy, remdesivir is employed as an antiviral agent that might lead to a faster recovery time but might not lower mortality significantly [139]. There are several trials using human plasma or hyperimmune globulin obtained from convalescent patients [140]. The clinically proven serine protease inhibitor camostat mesylate, which is active against TMPRSS2, partially blocked SARS-CoV-2 entry into Caco-2 and Vero-TMPRSS2 cells [113]. Chloroquine inhibits the production and release of TNF-α and IL-6, and thus may suppress the cytokine storm in critically ill patients. Chloroquine also interferes with terminal glycosylation of ACE2 and negatively influences receptor binding, possibly without interfering with ACE2 function and disturbing the ACE system [132]. Although chloroquine has been widely administered to treat COVID-19, to date, there is no evidence for a relevant clinical effect [141]. Tocilizumab, an anti-IL-6-receptor antibody, is an approved immunomodulator for rheumatoid arthritis and used in clinical trials to attenuate the cytokine storm including highly elevated IL-6 levels. Clinical studies have shown that tocilizumab is effective in treating severely ill patients [142].

The RBD in SARS-CoV-2 S protein seems the most likely target for the development of virus attachment inhibitors, neutralizing antibodies, and vaccines [12]. When blocking the RBD, the virus is hindered from binding to ACE2, hence impeding cell attachment and infection [10,143]. With SARS-CoV, immunization of mice with a RBD-based subunit vaccine elicited both antibody and cellular immune responses against the virus [143]. To date, no vaccines are approved against any human-infecting coronaviruses.

### 4.6. Control

Sudden increases in COVID-19 cases have paralyzed the health care system, even in developed countries. Without available vaccination, behavioural changes to prevent the spread of SARS-CoV-2 have been recommended or enforced by many countries. These include restriction of travel, social distancing, disinfection of surfaces, increased personal hygiene, screening, surveillance, and preventive quarantine. Mass gatherings are a risk factor for rapid spread and are currently suspended in many places [94]. Several countries have demonstrated that the spread of SARS-CoV-2 can be suppressed and controlled by implementing these measures [1]. The use of personal protective equipment is also used to control transmission and is especially important in health care settings.

For diagnostic purposes, real time PCR tests are most commonly used. However, the sensitivity is not optimal due to pre-analytical problems. For example, nasopharyngeal swabs yield a considerably higher positive rate than oropharyngeal swabs in the same cohort and a combination of both slightly increased sensitivity [144]. Sensitivity can also be increased by repeating swabs on a day-to-day basis or adding the analysis of stool samples, especially in children [95]. If PCRs are combined with antibody detection, the sensitivity increases significantly [128]. After all, controlling the spread of the highly contagious SARS-CoV-2 is the most important measure to mitigate the impact of the COVID-19 pandemic.

## 5. MHV as a Model for SARS-CoV and SARS-CoV-2

**Figure 1:**
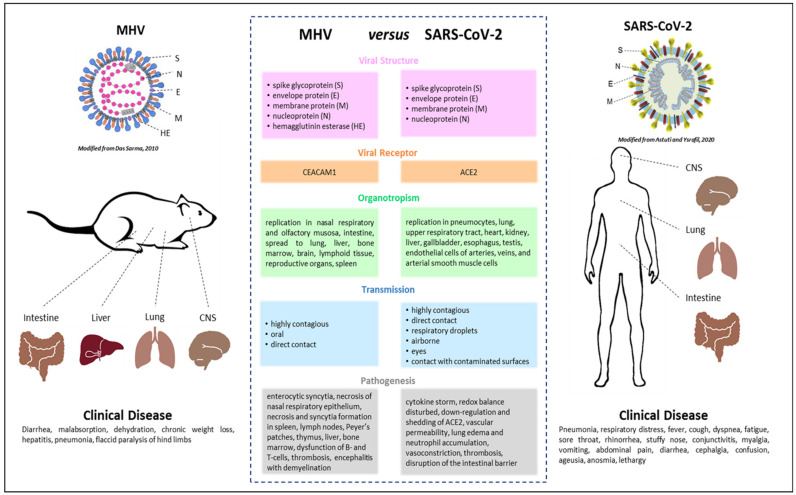
Summary of the main similarities and differences between the coronaviruses mouse hepatitis virus (MHV) and severe acute respiratory syndrome coronaviruses 2 (SARS-CoV-2). The images for MHV [155] and SARS-CoV-2 [156] in this figure were modified.

Seven coronaviruses cause human infection. Typically mild disease with common cold symptoms is observed in four of these, namely HCoV 229E, HCoV NL63, HCoV HKU1, and HCoV OC43. In contrast, MERS-CoV, SARS-CoV, and SARS-CoV-2 are zoonotic and, in addition to mild disease, also lead to severe respiratory illness and fatalities. The underlying immune mechanisms that contribute to the development of COVID-19 remain poorly understood partly because the initial outbreak is recent. The most intensely studied animal coronavirus is MHV, which induces a variety of conditions in mice, including respiratory, enteric, hepatic, and neurologic infections. Obtaining epidemiological information and molecular profiles of mice and humans, with MHV, or SARS-CoV-2, respectively, can help us in understanding the pathomechanisms of SARS-CoV-2, and in developing preventive strategies or clinical interventions. The present review provides comprehensive information pertaining to MHV and SARS-CoV-2, and the main points are summarized in Figure 1. One main focus of this work was to present information on MHV as a model for SARS-CoV-2 and the use of mouse models for elucidating the pathomechanisms of SARS-CoV-2.

MHV is an excellent model for studying the pathogenesis including tropism and virulence, as well as immune response to coronaviruses and was used as a model for SARS-CoV. Work with MHV only requires biosafety level 2 containment as opposed to biosafety level 3 containment when working with SARS-CoV and SARS-CoV-2. Interestingly, even though some coronaviruses are antigenically closely related they are biologically different [30], and pulmonary response in mice is mouse and virus strain-dependent [145,146]. Intranasal inoculation of BALB/c mice with MHV-3, MHV-A59, MHV-1, MHV-S, and MHV-JHM revealed that MHV-1, which is primarily pneumovirulent, produced a transient lung pathology most similar to SARS, which completely resolved by day 21. While C57BL/6J mice exhibited only a mild pulmonary disease, A/J mice developed severe progressive pulmonary disease by day 2 post-MHV-1 infection with 100% mortality within 7 to 10 days of infection. On the other hand, C3H/St mice developed intermediate susceptibility with 40% mortality by day 28. Similarly, after intranasal inoculation of susceptible A/J, C3H/HeJ, and BALB/c as well as resistant C57BL/6 strains of mice with MHV-1 both A/J and C3H/HeJ mice exhibited enhanced weight loss and clinical illness compared to BALB/c and C57BL/6 mice [147]. C3H/HeJ mice, which naturally lack the toll-like receptor 4 [148], showed increased morbidity, mortality, and severe pulmonary disease compared to C3H/HeN mice [147]. Intranasal inoculation with MHV-1 produced lethal pneumonitis in A/J mice [149] and acute lung injury in 129/SvEv/C57BL6/J mice [150].

Another report showed that MHV-A59 replicated in the lung and induced acute pneumonia and severe lung injuries in both young and old C57BL/6 mice, which closely mimicked ARDS by SARS-CoV and MERS-CoV in human lungs [151]. Also, since SARS-CoV-2 infection may produce neurological features including central nervous system (CNS) injuries [152,153] MHV-A59 and MHV-JHM may be used as models to decipher the mechanisms of virus entry into the CNS and the resulting immune response.

Notably, MHV-1 replicated well to similar levels in the lung in all strains of mice inoculated [145,147]. Tissue destruction occurs due to viral replication but severe lung damage is mainly immunopathological in nature, correlating better to the elevated inflammatory responses than to viral replication in the lung [154]. Therefore, inhibition of inflammatory responses is important in protecting the lung from injury.

Very small changes in coronavirus proteins can greatly affect tropism and virulence, which do not depend only on the spike protein [157], but also on a combination of the structural protein M and the nonstructural replicase-associated proteins nsp1 and nsp13, these proteins being conserved among β-coronaviruses [158]. Less type 1 IFN was produced by A/J mice than in resistant strains of mice [149,159]. A previous report show that the lack of signaling by the CD200 receptor, a myeloid receptor [160] which is expressed on macrophages, granulocytes, dendritic cells, T cells, B cells, and NK cells [161,162], strongly enhances type I IFN production and viral clearance, thereby improving the outcome of MHV infection, particularly in female mice. MHV clearance is dependent on TLR7-mediated type I IFN production. Sex differences in TLR7 responses were reported for humans [163]. The total number of IFNγ-producing CD4 T cells was significantly increased in C3H/HeJ mice compared to C3H/HeN mice. Both CD4 and CD8 T cells contribute to the MHV-1-induced disease [159,164] since depletion of either subset ameliorates morbidity and mortality. Also, a significant increase in the total number and frequency of T regulatory cells may aid in modulating the CD4 and CD8 T cells [165]. Serum cytokines and chemokines were markedly elevated in susceptible A/J mice [145].

## 6. Mouse Models for Infection with SARS-CoV and SARS-CoV-2

Different animal models including mice, ferrets, Syrian hamsters, and primates [166,167,168,169,170,171] are used to study SARS-CoV-2. An ideal animal model should reproduce viral replication, clinical signs and symptoms, pathogenesis, and immune response. Due to the interspecies differences, no single animal model for SARS perfectly recapitulates the phenotype. Being able to conduct research with small animal models is desirable, since it can help us understand the pathogenesis and speed up development of new therapies and vaccines. There are various advantages of mice, including their small size, rapid reproduction, accessible technology for genetic modification, and comparability based on genetically identical cohorts. Notably, the use of animal models for SARS-CoV-2 infection is challenged by the requirements of biosafety level 3 containment and restricted license to conduct effective research. Since SARS-CoV-2 does not bind to the murine ACE2 receptor there are basically two ways of developing a mouse model; either by altering the host or the virus.

In contrast to SARS-CoV-2, SARS-CoV is able to infect wild type mice. After nasal inoculation of 4- to 6-week-old BALB/c mice the virus replicated in the respiratory tract and was cleared within one week. Neutralizing antibodies were produced and prevented viral replication in naïve mice by prior transfer of immune serum from convalescent mice. Notably, mice did not show any symptoms. Furthermore, pathological lung changes were restricted to mild and focal peribronchiolar mononuclear inflammatory infiltrates. Viral antigens and nucleic acids were only located in bronchiolar epithelial cells [172]. Also, five- to six-week-old C57BL/6 mice supported transient nonfatal systemic infection with SARS-CoV in the lung, which disseminated to the brain. It was suggested that a highly effective innate antiviral response in the lung was primarily responsible for viral clearance. In contrast, adaptive cellular immunity and natural killer cells played only a minor role [173]. Another study using 129SvEv mice showed that SARS-CoV infection resulted in self-limited bronchiolitis but progressed to severe pulmonary inflammation in Signal Transducer and Activator of Transcription 1 (STAT1) knockout mice, which are resistant to the effect of interferons, undermining the importance of the interferon response [174]. Taken together, due to the differences in pathogenesis to human disease the use of wild type mice in SARS-CoV research was limited.

Mouse models have been widely used to investigate determinants of lung development, aberrant alveolarization, chronic lung diseases, and inflammatory response [175,176]. Human and mouse lung development progresses through five successive stages. While the sequence of the stages are identical in both species, the timing during these periods varies. For example, lungs of term infants are in the alveolar phase of lung development at birth, whereas mice are in the saccular stage [176,177,178]. This aspect is important when attempting to model age-dependent lung diseases [179,180,181]. To overcome this limitation of mouse models, novel methodologies need to be developed using humanized mouse models such as models with natural human target cells [182].

The fact that the prevalence of COVID-19 is much higher in adults than in children indicates that aging-related processes in the lung increase the susceptibility for an infection with SARS-CoV-2. This is most likely due to immunosenescence. The concept of aging processes in the immune system coupled with changes in cytokine responses has also been referred to as inflammaging and could adversely modify and exaggerate immunological responses following viral infection [183,184]. Notably, the majority of murine studies investigating immune response, pathomechanisms of viral infection, as well as acute and chronic lung diseases use animals that are rarely older than six months. A recent study [185] investigated the lung cell-specific changes in young and old mice and proposed a deregulated control of epigenetic processes and cell metabolism in lung cells, as well as lung matrix remodeling in aging lungs. These age-related dynamics could facilitate virus adhesion and entry in the respiratory system in older humans and mice. Therefore, the age-dependent dynamic of organ physiology, matrix remodeling, cell homeostasis, and immune response need to be considered in COVID-19 research.

In contrast to the mild pathological changes seen in SARS-CoV infection in young wild type mice, infection of 12- to 14-month-old BALB/c mice was associated with increased viral replication in the lungs, infection of pneumocytes, interstitial pneumonia, clinical illness, and weight loss [186]. Thus, several models of SARS employing aged mice were established. Compared to young mice, aged BALB/c mice showed elevated levels of IFN-α, IFN-γ, and TNF-α early in infection [186]. Another study with 12- to 14-month-old BALB/c mice showed that depletion of CD4 T cells lead to an aggravated immune-mediated interstitial pneumonia, delayed clearance of SARS-CoV, reduced neutralizing antibody and cytokine production, as well as reduced pulmonary recruitment of lymphocytes [187]. The depletion of CD8 cells had no effect. This result points to an important role of CD4 cells in viral clearance in aged mice.

Various genetically engineered mouse lines were developed for studies with SARS [188,189,190]. Mice transgenic for the expression of hACE2 (hACE2-transgenic mice) were infected by SARS-CoV and replication occurred in the lung [188]. However, SARS-CoV also spread to the brain and the infection finally resulted in death due to CNS failure and not as a result of SARS [79]. Another study also found high virus titers in the lungs and in the brains [191]. Viremia occurred and lower virus titers were also detected in other organs. Mortality was 100%, most likely due to CNS failure. Notably, the same study used a second transgenic line with less abundant expression of hACE, which did not develop relevant CNS infection and recovered completely. Nevertheless, due to a different pathomechanism, leading to a fatal outcome, hACE2-transgenic mice are generally not regarded as an optimal model for the study of SARS. Recently, transgenic mice were also generated for SARS-CoV-2 research [192]. SARS-CoV-2 infection lead to interstitial pneumonia with diffuse lesions. However, the pathogenicity of SARS-CoV-2 compared to SARS-CoV was mild in these transgenic mice. Another mouse model expressing human ACE2 (hACE2) was created by using CRISPR/ Cas9 knockin technology. Both young and aged hACE2 mice showed high viral loads in lung, trachea, and brain. The viral loads in the lung were higher than in the previously described model. Interstitial pneumonia and elevated cytokines were aggravated in aged hACE2 mice. Nevertheless, there were no fatalities [193].

A different approach to alter the host is based on the transduction with an adenoviral vector, which leads to transient expression of hACE2 [194,195]. Prior to infection with SARS-CoV-2, mice were inoculated with a replication-deficient adenovirus (Ad5-hACE2). This approach is faster than the generation of transgenic mice and limits the expression of hACE2 to the respiratory system. Ad5-hACE2-sensitized mice infected by SARS-CoV-2 showed weight loss, high viral replication in the lungs, and severe pulmonary pathology [194,195]. Cytokine and chemokine responses to SARS-CoV-2 infection were similar to those observed in humans [195]. However, no lethalities were reported. Some therapeutic options were evaluated including patient-derived convalescent plasma, neutralizing monoclonal antibodies, the interferon I inducer Poly I:C, and the antiviral remdesivir, which were effective in these mice [194,195].

As already mentioned, the infection of young mice results only in mild disease. By serial passage in the murine respiratory tract it was possible to develop mouse-adapted virus strains that lead to severe disease. Mouse-adapted SARS-CoVs were created by several groups and have been used broadly [196,197,198]. Infection with the mouse-adapted virus MA15 rapidly lead to high viral replication in lungs, viremia, and extrapulmonary manifestations [196]. Lymphopenia, neutrophilia, and pathological changes in the lungs were observed. The mice eventually died from the systemic viral infection coupled with extensive destruction of pneumocytes and ciliated epithelial cells. Notably, the pulmonary damage does not comprise alveolar edema and hyaline membrane formation, as observed in patients and older mice infected with SARS-CoV. It was suggested that the mice did not survive long enough for development of diffuse alveolar damage. Therefore, the mechanisms leading to death during an infection with MA15 differs from SARS-CoV. It is mainly based on the rapid progression of the infection, which results in 100% mortality, and was also lethal for young mice. To evaluate the reasons for the severe disease in young mice, MA15 mutations were further analyzed [199], showing that foremost mutations in the S protein and partly in the replicase nonstructural protein nsp9 were essential to enable infection of young mice. A similiar observation was made with the mouse-adapted virus v2163 [198]. It was suggested that mutations in the S protein led to changes in binding properties and increased virulence in young mice. Therefore, mutations in the RBD of the S protein contribute to age-related disease severity.

A different mouse-adapted virus based on a mouse-passaged Frankfurt 1 isolate of SARS-CoV maintained age-dependent severity. Infection lead to severe respiratory illness in all adult (6-month-old) mice with a mortality rate of 30 to 50% [197]. In contrast, young mice (4-week-old) did not develop severe disease. Moreover, severely ill adult mice developed pulmonary edema and diffuse alveolar damage. As observed in humans, a cytokine storm with macrophage and neutrophil infiltration preceded these pathological changes. Adult mice showed higher levels of IL-1α, IL-1β, TNF-α, and IL-6 than young mice, the latter having higher levels of IFN-γ, IL-2, IL-10, and IL-13. This different cytokine response is consistent with the human disease and highlights it as a key driver for age-dependent severity, the insufficient release of IFN-γ in adult mice being the main difference. Treatment of adult mice with intraperitoneal injection of IFN-γ resulted in milder histopathological changes and protected adult mice from a fatal outcome.

Recently, a mouse-adapted strain of SARS-CoV-2 was created [200] by remodeling the S and mACE2 binding interface via reverse genetics, a technology that was first developed for targeted recombination of MHV-A59 [201], resulting in a recombinant virus (SARS-CoV-2 MA) that utilizes mACE2 for cell entry. SARS-CoV-2 MA is able to replicate in the upper and lower airways of young adult and aged BALB/c mice. As in the previously described models, disease is more severe in aged mice. In contrast to the human disease, the extent of viral replication in the upper airways is less than that observed in the lung. Prophylactic and therapeutic administration of IFN-λ-1a resulted in diminished replication of SARS-CoV-2 MA in mice. Furthermore, serum from S vaccinated mice was able to neutralize SARS-CoV-2 MA.

## 7. Conclusions and Future Directions

This review provides a comprehensive overview of MHV and SARS-CoV as possible murine surrogate models to understand, decipher, and ultimately use the pathomechanisms and viral characteristics of SARS-CoV-2 as therapeutic approaches. For this purpose, we first described MHV and SARS-CoV-2 in detail with respect to viral strains, host specificity, organotropism, transmission, pathogenesis, as well as clinical disease, mortality, therapy and control in order to clearly highlight the similarities and differences. The question arose concerning the extent to which murine studies using MHV may serve as surrogate models for SARS-CoV-2 to understand the viral biology and to decipher new preventive and therapeutic strategies for COVID-19, a new devastating disease. Based on the diversity of coronaviruses, we also provide information on SARS-CoV and MERS-CoV.

Various lessons can be learnt from these well-established and characterized murine models. Similarities between murine infection with MHV and human infection with SARS CoV-2 include their affinity for the olfactory system (MHV-A59) and pulmonary system (MHV-1), their spread into the brain causing neurologic symptoms (MHV-A59, MHV-JHM), development of microthrombi in the liver (MHV) or the brain (SARS-CoV-2), their high virulence associated with an inability to induce a robust T-cell response, their ability to modify immune response, RNA persistence in the gastrointestinal tract (MHV, SARS-CoV-2) and, depending on the strain, also in the brain (MHV, SARS-CoV). Major differences between MHV and SARS-CoV-2 include the viral receptor (CEACAM1a without co-receptors vs. ACE2 with co-receptors, respectively) as well as some aspects of organotropism and clinical disease. MHV infects only mice while SARS-CoV-2 infects different species including humans, hamsters, ferrets, cats, nonhuman primates.

The extensive comparison between MHV and SARS-CoV-2 emphasizes the fact that while MHV can provide some insight into the viral biology of SARS-CoV-2, it does not fully recapitulate the complexity of this new virus, for example, in terms of virus entry or recognition. Therefore, MHV-infected mice may not offer a perfect model for COVID-19 but definitely a surrogate model. These findings show the enormous importance of the precise characterization and understanding of murine virus models.

Further key disease modifiers need to be taken into account when investigating the biology of MHV and SARS-CoV-2. The investigation of mechanisms which determine heterogenic organotropism of various MHV strains and which protect certain mouse strains from clinical manifestation could provide new and important insights into virus-host interaction. This knowledge may eventually be transferred to SARS-CoV-2. The fact that COVID-19 particularly affects older people, while children and adolescents are more protected, highlights that age is a factor in the susceptibility of the host to SARS-CoV-2. Notably, this review provides strong evidence that both gender and age are central in the manifestation of clinical signs, course of the disease, and mortality in mice and humans. It is crucial to decipher the mechanisms which protect younger people to ultimately develop new therapeutic strategies. Currently, the mechanisms directing the processes of the aging immune system and determinants of susceptibility to SARS-CoV-2 are elusive. Similar differences in cytokine response to SARS-CoV and SARS-CoV-2 infections in mice and humans highlight the important pathomechanistic role of the innate immune system in determining the severity of the disease. However, the exact pathways have yet to be unveiled.

As depicted in this review, a lot of progress has been made by conducting research with wild type viruses and wild type mice. In recent years, new strategies comprising the modification of the host and virus strains by genetic engineering and other techniques lead to new insights into virus-host interaction. Regarding the COVID-19 pandemic, we observe an increased speed of knowledge generation and certainly mouse models will continue to play an important role in this fast paced scientific world. While the advantages and important insights into virus biology using murine MHV models have been recognized, the limitations of this surrogate system require new strategies. To this end, further basic research on coronaviruses and new models including humanized mouse models and mouse-adapted viruses are urgently needed to elucidate the pathomechanism of SARS-CoV-2 infections, enabling the development of new vaccines and therapies.
